# MXenes-integrated microneedle combined with asiaticoside to penetrate the cuticle for treatment of diabetic foot ulcer

**DOI:** 10.1186/s12951-022-01468-9

**Published:** 2022-06-07

**Authors:** Pei Wang, Yun Wang, Yang Yi, Yan Gong, Haoran Ji, Yuci Gan, Fei Xie, Jinchen Fan, Xiansong Wang

**Affiliations:** 1 Department of Thoracic Surgery, Shanghai Key Laboratory of Tissue Engineering, Shanghai Ninth People’s Hospital, Shanghai Jiao Tong University School of Medicine, Shanghai, 200011 China; 2 School of Materials and Chemistry, University of Shanghai for Science and Technology, Shanghai, 200093 People’s Republic of China; 3 Faculty of Environment and Life, Beijing University of Technology, Beijing, 100124 People’s Republic of China

**Keywords:** Microneedle, MXenes, Asiaticoside, Angiogenesis, Diabetic foot ulcer

## Abstract

Patients with diabetic foot ulcers usually suffer from inefficient epithelisation and angiogenesis accompanied by chronic wound healing. Diabetic foot ulcers remain a major challenge in clinical medicine; however, traditional treatments are incapable of transdermal drug delivery, resulting in a low drug delivery rate. We report the development of Ti_2_C_3_ MXenes-integrated poly-γ-glutamic acid (γ-PGA) hydrogel microneedles to release asiaticoside (MN-MXenes-AS). Asiaticoside was loaded into PGA-MXenes hydrogel to facilitate cell proliferation while regulating angiogenesis. The characterisation and mechanical strength of the microneedles were investigated in vitro, and the wound-healing efficacy of the microneedles was confirmed in diabetic mice. MXenes significantly improved the mechanical strength of microneedles, while γ-PGA hydrogels provided a moist microenvironment for wound healing. Mice treated with MN-MXenes-AS demonstrated obvious improvements in wound healing process. We successfully fabricated an MXenes-integrated microneedle that possesses sufficient rigidity to penetrate the cuticle for subcutaneous drug delivery, thereby accelerating diabetic wound healing. We demonstrated that MN-MXenes-AS is effective in promoting growth both in vivo and in vitro. Collectively, our data show that MN-MXenes-AS accelerated the healing of diabetic foot ulcers, supporting the use of these microneedles in the treatment of chronic wounds.

**Graphical Abstract:**

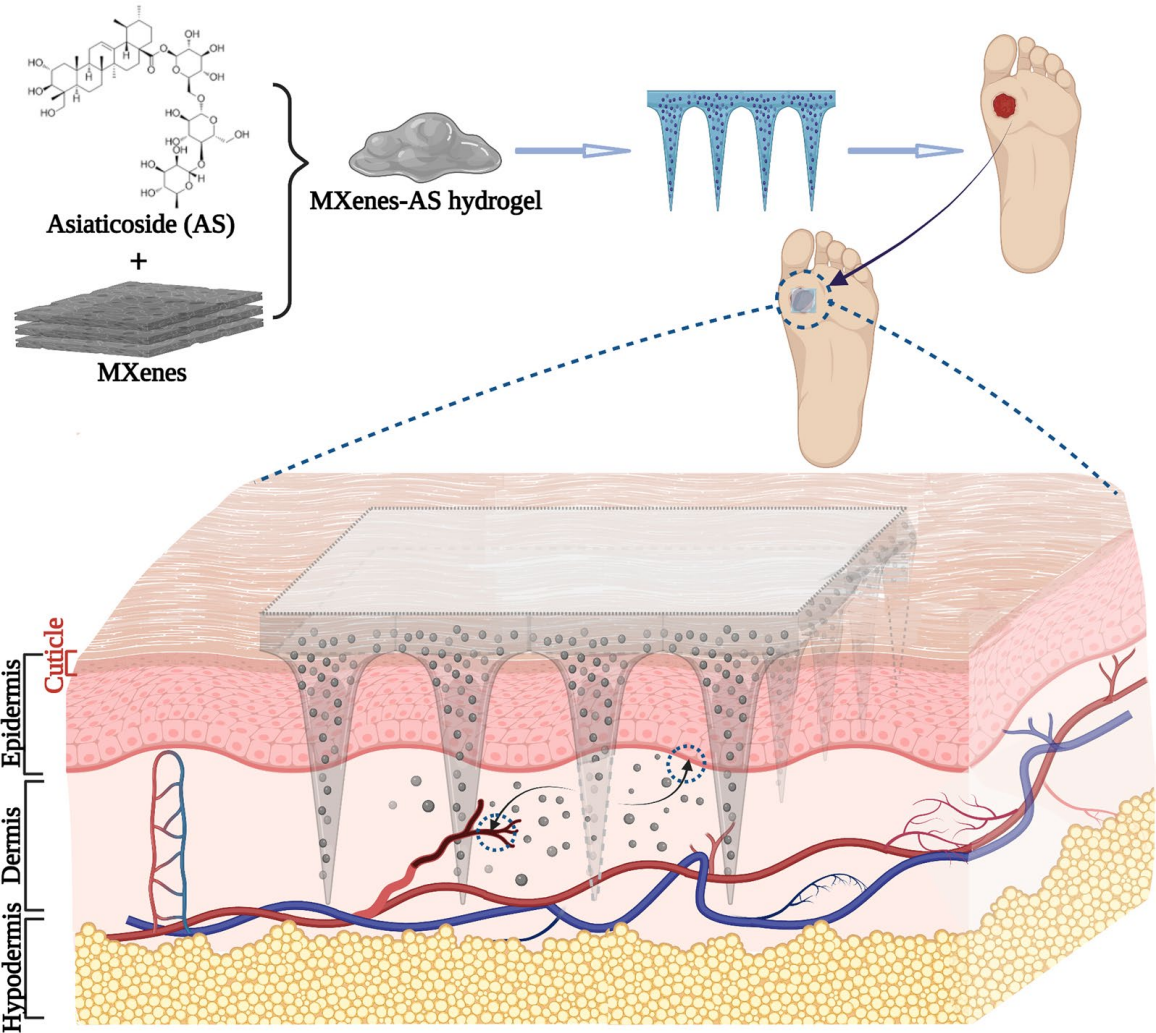

**Supplementary Information:**

The online version contains supplementary material available at 10.1186/s12951-022-01468-9.

## Introduction

Without an efficient and transdermal drug delivery system, patients who have skin disorders of various causes [1] tend to experience incomplete or improper wound healing [2]. Diabetic foot ulcer (DFU) resulting from decreased neurovascular response [3] and multi antibiotic-resistant bacterial infection [4] are an example of chronic wound healing. DFUs are accompanied by high morbidity and mortality [5] and can lead to limb amputations [6] and hospitalization [7]. Unfortunately, most treatments for diabetic skin disorders, including the application of acellular dermal matrix (ADM) [8, 9], electrospun nanofiber [10], are administered by smearing and are incapable of efficiently delivering drugs through the cuticle. Thus, to promote skin regeneration in patients with diabetes mellitus, it is necessary to develop a better drug delivery system.

Microneedles are important tools that have been applied in multiple medical fields to deliver a variety of substances, such as contraceptives [11, 12], insulin [13], glucagon [14] and stem cell [15]. As a transdermal delivery system, microneedles can penetrate the cuticle and delivering drugs deeper and faster [16], it also possesses excellent biodegradability with minimal pain [17] (or painless when the length is within 1 mm [18]). In combination with different materials [19, 20], microneedles can be used for various purposes.

Quick-dissolving and hydrogel-forming microneedles have become increasingly popular [18]. Traditionally, high-molecular compounds have been used to fabricate basic hydrogel such as polylactic-co-glycolic acid (PLGA) [11, 15, 17]. However, PLGA has low biodegradability and takes almost two weeks to completely dissolve. Biopolymers, such as hyaluronic acid, a component of extracellular matrix (ECM) [21–23], possess better biocompatibility and biodegradability, making them more suitable for use in microneedles. In our previous study, we successfully demonstrated the efficacy of a poly-γ-glutamic acid (γ-PGA) hydrogel [24] and γ-PGA-based microneedles (MN-PGA) in promoting diabetic wound healing [25]. The hydrophilic γ-PGA polymer is superior to hyaluronic acid because of its excellent biodegradability, biocompatibility, edibility. Compared with PLGA, γ-PGA possesses better water solubility [26], as it can be dissolved within 20 min. In addition, γ-PGA provides a moist microenvironment for healing wounds and simulate glycosaminoglycans biofunction [27], including the delivery of bioactive factors and proteins, metabolites discharge.

However, the drug delivery efficacy of microneedle is restricted by its dissolution time and mechanical strength, which may lead to incomplete insertion [17]. As expected, γ-PGA-based MN usually has rather insufficient mechanical strength to fully penetrate the cuticle. To resolve this problem, we combined γ-PGA-based microneedles with Ti_2_C_3_ MXenes (MN-MXenes). In the biological field, MXenes, novel 2D nanosheets, are mostly used in synergistic chemotherapy [28] for cancer treatment, bioimaging [29], and ultrasensitive detection [30]. MXenes exhibit excellent mechanical properties [31] and possess a remarkable drug-loading capability of over 200% [32]. In addition, MXenes possess great biocompatibility without any cytotoxicity to normal cell lines [33]. Thus, in this study, we sought to use MXenes as drug-loading nanosheets and as special approaches to further improve the mechanical strength of γ-PGA-based microneedles.

Asiaticoside (AS), an organic compound, is considered the main therapeutic agent for promoting cell proliferation and monitoring angiogenesis during wound healing in patients with diabetes. Asiaticoside (AS) has demonstrated efficacy in facilitating epithelialization [34], inducing osteogenic differentiation [35] and osteoclastogenesis [36], and promoting fibroblast proliferation and angiogenesis [37]. Previous work has demonstrated that AS has a biphasic effect: it not only promotes the angiogenesis in the inflammation stage, but also weakens the angiogenesis during the remoulding stage [38], which dynamically impacts wound closure [37–40]. Due to its effect on wounds, AS can be applied as a practical compound to promote chronic wound healing.

**Scheme 1:**
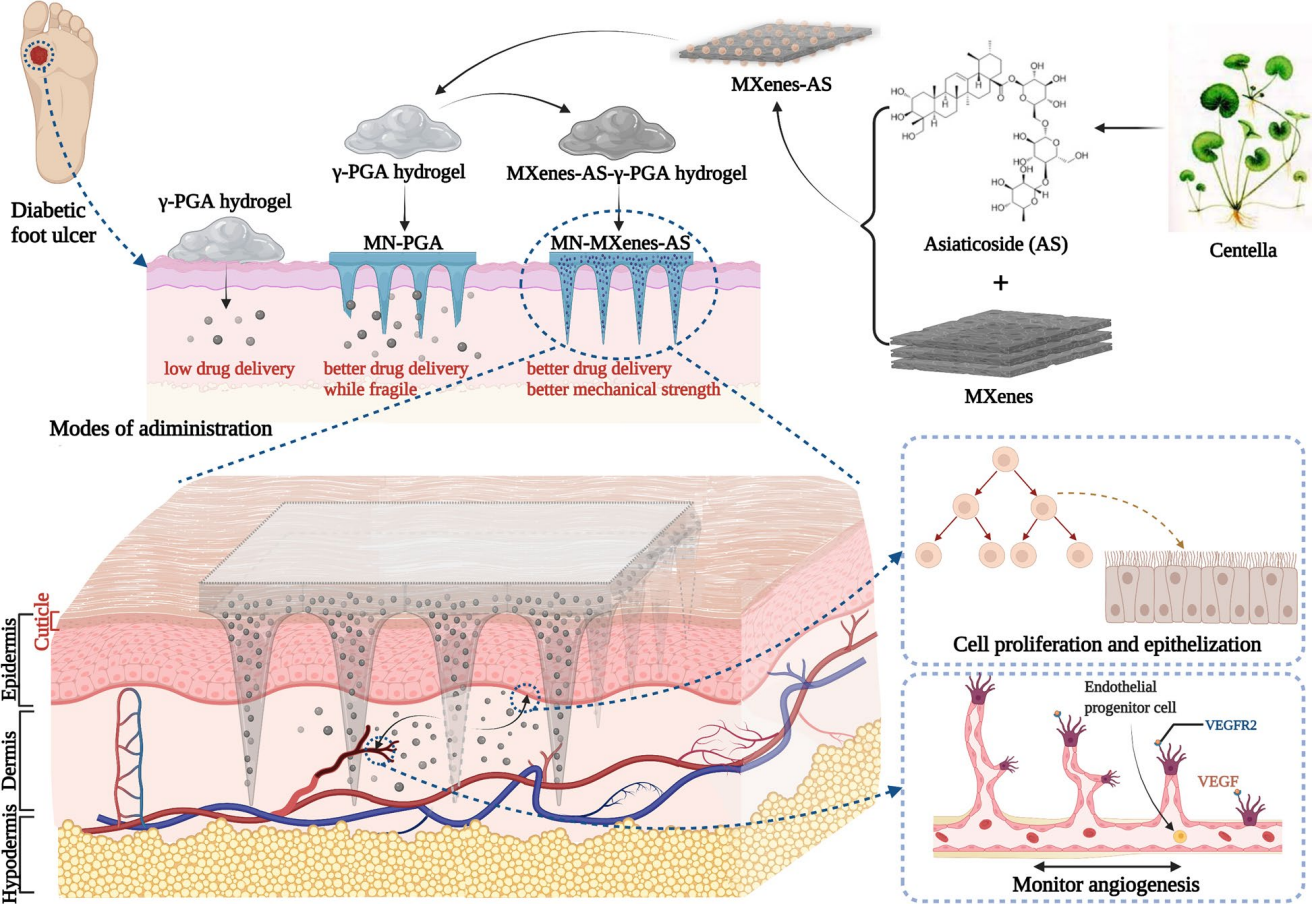
Schematic illustration of MXenes-based microneedle patch (denoted as MN-MOF-GO-Ag) for accelerating diabetic wound healing (Image was created with BioRender)

Herein, we successfully manufactured a MXenes-integrated microneedle to slowly release AS (MN-MXenes-AS) to accelerate chronic wound healing (Scheme 1). The γ-PGA hydrogel, with MXenes-AS homogeneously distributed (Additional file 1: Fig. S1), dissolved in body fluids within 20 min and led to AS release. The use of MXenes as a drug-loading system simultaneously improved the mechanical strength of the microneedles to allow penetration of the cuticle for subcutaneous drug delivery and extended the release time of AS. This novel MN-MXenes-AS would be a promising way to treating DFUs.

## Results

### Synthesis and characterization of MN-MXenes-AS

**Fig. 1:**
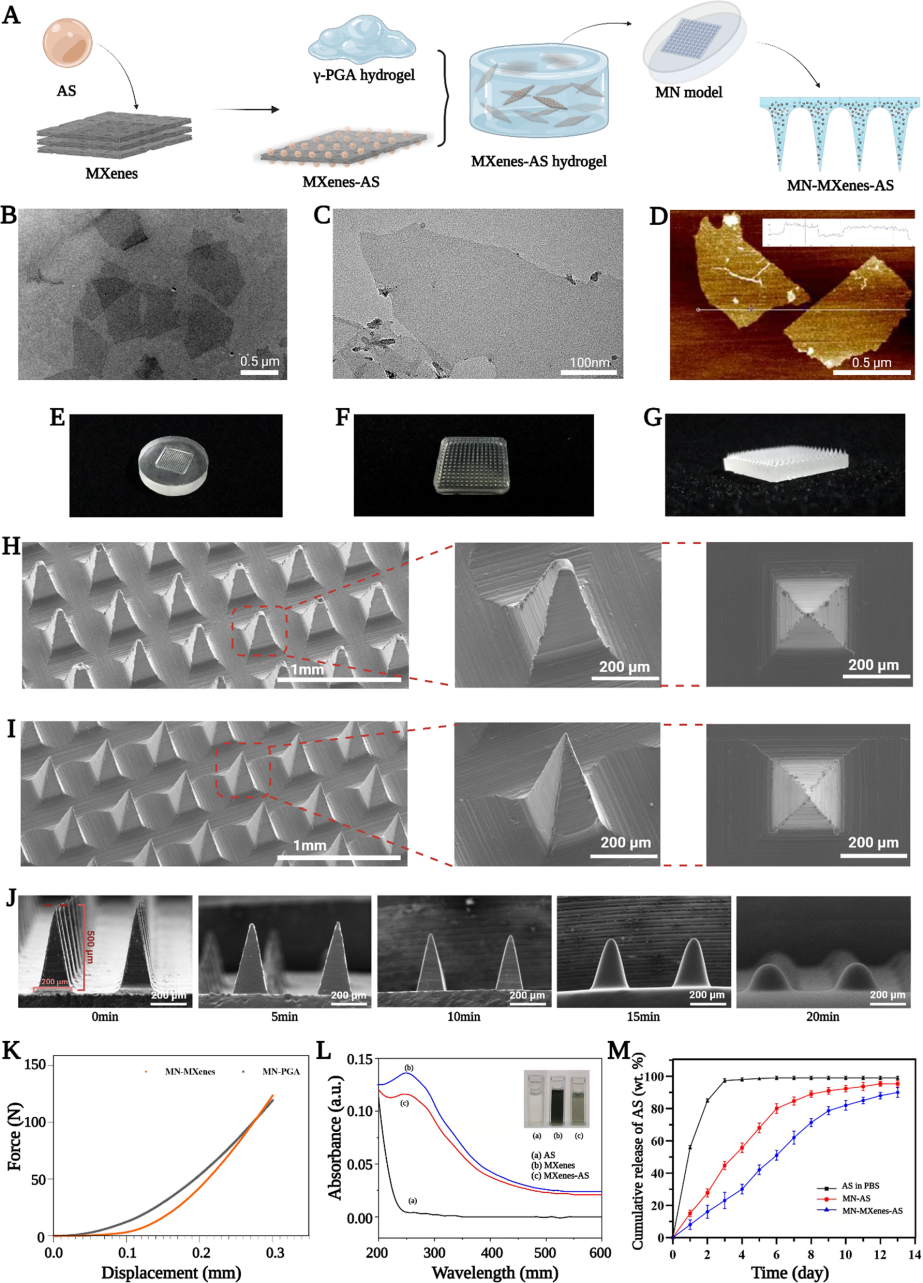
Synthesis and characterization of MN-MXenes-AS. **A** Schematic illustration of the synthesis of an MN-MXenes-AS. **B**, **C** The TEM images of MXenes. **D** The AFM image of MXenes. **E** Photographic image of PDMS microneedle master mold. **F**, **G** Profile and isometric photographic images of an MN-MXenes-AS. **H** SEM images of an MN-PGA patch with different angles. **I** SEM images of an MN-MXenes-AS patch with different angles. **J** SEM images of MN after moisture absorption at different time points (75% humidity box, room temperature). **K** The comparison between the mechanical strength of MN-PGA and MN-MXenes-AS. **L** Absorption spectrum of AS, MXenes and MXenes-AS. **M** Percentages of the released AS from an MN-AS, MN-MXenes-AS in the presence of PBS or from AS in PBS

MXenes were loaded with AS then immersed into the γ-PGA hydrogel to facilitate the formation of the MXenes-AS hydrogel, which had a darker color than γ-PGA hydrogel. The MXenes-AS hydrogel was then added to a microneedle mould and dried to produce a microneedle with suitable mechanical strength (Fig. 1A).

The MXenes used in these experiments were single-layer nanosheets. Transmission electron microscopy and atomic-force microscopy were used to characterize the MXenes (Fig. 1B–D) and heights of MXenes were controlled to within 2 nm. Fig. 1E–G show the general morphology of the microneedle mould, which possesses 10× 10 arrays. Each microneedle was in the shape of a rectangular pyramid with dimensions of 200 μm ×200 μm × 500 μm (W ×L × H).

MXenes were integrated into the microneedle for simultaneous drug loading and mechanical strength improvement. We used scanning electron microscopy (SEM) and mechanical testing instruments to compare the micro-phenotypes and mechanical strengths, respectively, of MN-PGA and MN-MXenes-AS. MN-MXenes-AS exhibited better morphology, was easier to preserve after being demoulded, and had higher mechanical strength than MN-PGA (Fig. 1H, I, K). SEM images of MN-PGA and MN-MXenes-AS under different humidity or temperatures are presented in Additional file 1: Fig. S3, S4 and S5. Microneedles used in clinic medicine should not only have sufficient mechanical strength, but must also possess high biodegradability. Thus, we tested the degradation time of MN-MXenes-AS. As shown in Fig. 1J, MN-MXenes-AS dissolved within 20 min (at 75% humidity, 25 ℃), which satisfies the requirement for this application.

In addition to the morphology, performance measurements are also important. To determine whether AS was fully combined with MXenes, the absorption peaks of AS, MXenes, and MXenes-AS were measured. We observed different peaks between AS or MXenes alone and MXenes-AS, indicating that AS and MXenes had successfully combined (Fig. 1L).

We hypothesized that microneedles containing MXenes would release AS over a longer period of time. To testify our hypothesis, the release of AS immersed in different materials was tested for 12 days. As shown in Fig. 1M, MN-MXenes-AS released AS for a longer amount of time than AS in PBS.

### Fluorescent assay for the insertion depth of MN

**Fig. 2:**
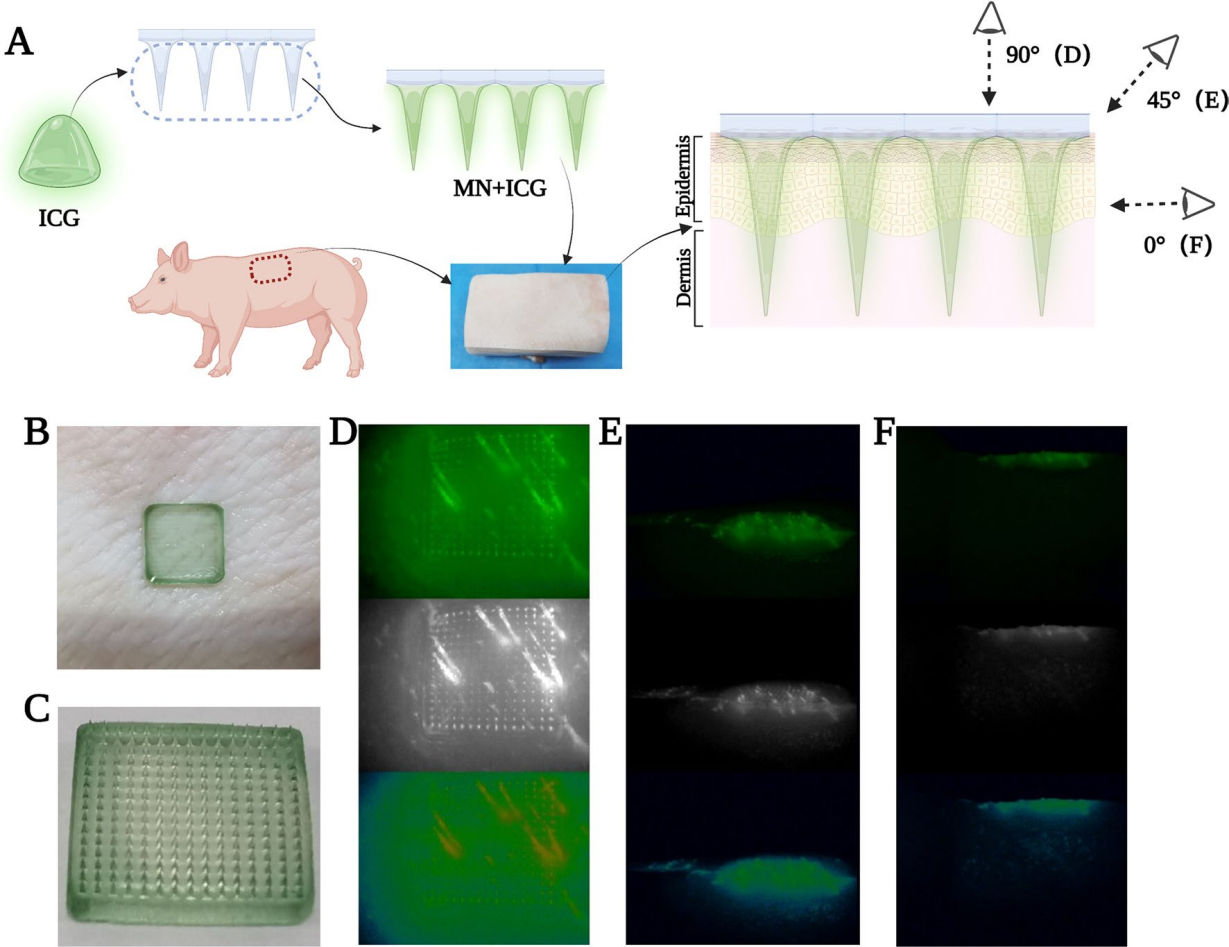
Fluorescent assay for the insertion depth of MN. **A** Schematic illustration of MN loaded with ICG fluorescent assay and insertion of it. **B** Picture of MN inserted into pig skin. **C** Top-down photographic images of an ICG loaded MN. **D–F** The top-down and side fluorescent images of an ICG loaded MN

Microneedles with MXenes have better morphology and mechanical strength than those without MXenes; however, this does not guarantee that MN-MXenes-AS will penetrate the cuticle. The skin of a pig has a density and cuticle thickness similar to human skin. We used a piece back skin from an adult pig to test the mechanical strength of MN-MXenes-AS in vitro (Fig. 2A). To make the result more intuitive, the microneedle was inserted into the skin (Fig. 2B) after it was loaded with the fluorescent assay indocyanine green (ICG) (Fig. 2C). The back layer of MN was removed after the tips were dissolved. Images were taken with an HD Fluorescent Endoscopy System (OptoMedic 2100 Series) at 0°, 45° and 90° angle to the horizontal (Fig. 2D–F). The skin tissue became fluorescent, indicating that ICG had been delivered through the cuticle. Therefore, MN-MXenes-AS had sufficient mechanical strength to penetrate cuticles.

### In vitro cell viability, migration and cytotoxicity of MN-MXenes-AS

**Fig. 3:**
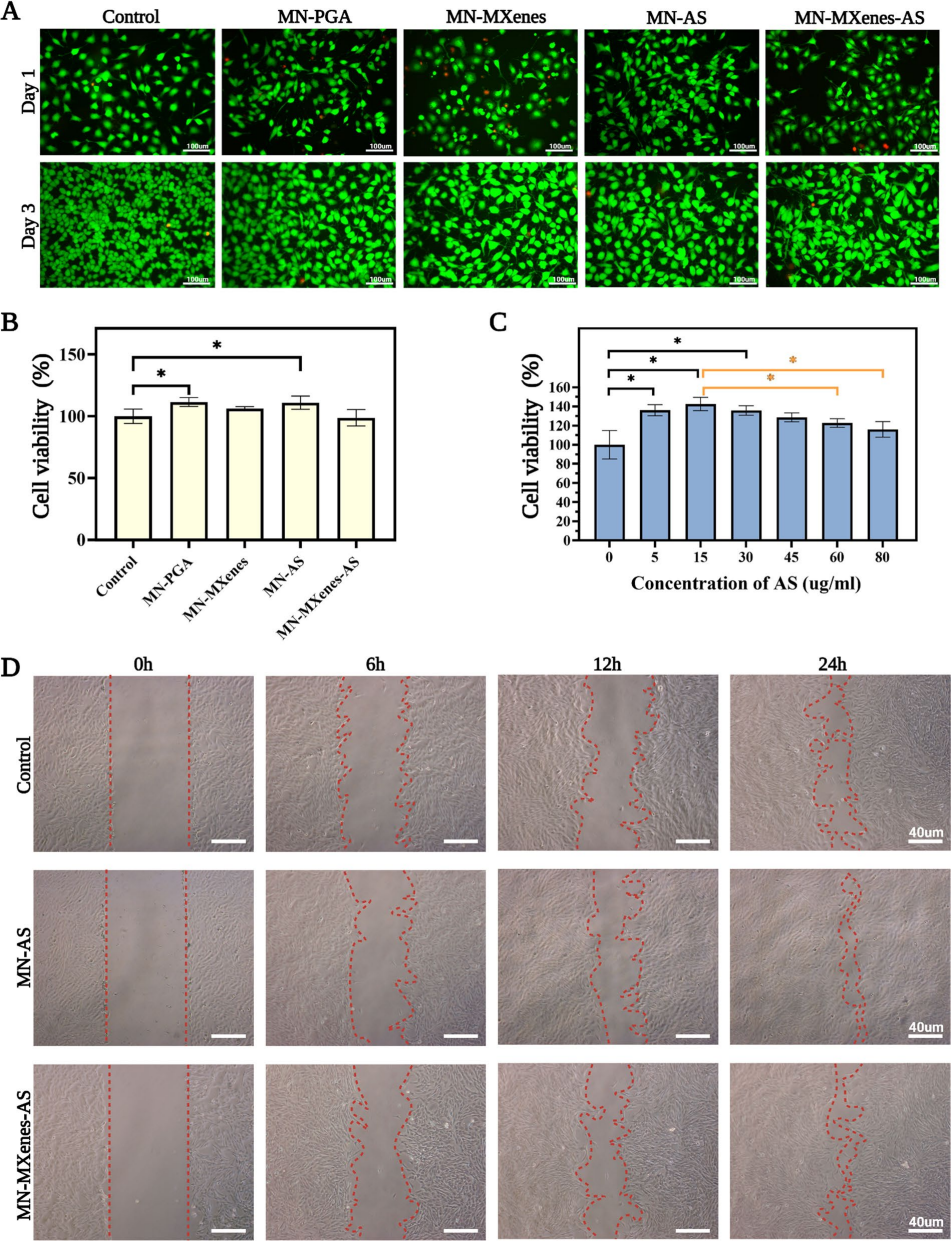
In vitro cell viability, migration and cytotoxicity of MN-MXenes-AS. **A** Confocal images of Living/Dead cell double staining of HUVEC after cocultured with different MN for 24 h and 72 h. **B** The cell viability of fibroblast cocultured with either MN-PGA, MN-AS, MN-MXenes, MN-MXenes-AS extract, or no materials for 72 h. **C** Cell viability of different groups after incubation with different concentration of AS for 72 h. **D** Photographic images of the migration of fibroblast incubated in normal cell culture medium (control) or cell culture medium containing AS or MN-MXenes-AS extract for 0 h, 6 h, 12 h and 24 h

Biomaterials are usually demanded to have growth-promoting properties without obvious cytotoxicity. Therefore, we used human umbilical vein endothelial cells (HUVECs) and fibroblast to test cytotoxicity. HUVEC were divided into five groups and co-cultured with either MN-PGA, MN-AS, MN-MXenes and MN-MXenes-AS extract, or no extract for 1 or 3 days. Confocal images were taken after the cell were stained with the living/dead cell double staining kit. Fig. 3A summarizes the most representative images from different groups. HUVECs grew normally when co-cultured with different types of microneedles, indicating that the MN-MXenes-AS patch was not cytotoxic to HUVECs. Fibroblasts exposed to MN-MXenes-AS were counted using cell counting kit-8 (CCK-8) and also indicate did not exhibit cytotoxic effects, although AS had a statistically significant effect on cell growth (Fig. 3B).

To test which concentration of AS benefit cell most, fibroblasts were cocultured with different concentration of AS solution (0, 5, 15, 30, 45, 60, and 80 μg/ml); no cytotoxic effects were detected at concentrations up to 80 μg/ml (Fig. 3C). Meanwhile, the viability of cells co-cultured with AS at concentrations of 5–30 μg/ml for 72 h was significantly higher (p < 0.05) compared with control group. The mechanism underlying AS promoting growth was examined using in vitro cell migration experiments. Fibroblast were seeded in 6-well plates and divided into three groups. A scratch was applied to the middle of each well, after which fibroblasts were co-cultured either with AS solution (15 μg/ml), MN-MXenes-AS extract or no materials (control group). Representative images were taken with a microscope after cells being cocultured for 0, 6, 12 and 24 h and presented in Fig. 3D. We found that AS solution and MN-MXenes-AS extract both improved the migration of fibroblasts.

### In vivo wound healing evaluation with diabetic mice

**Fig. 4:**
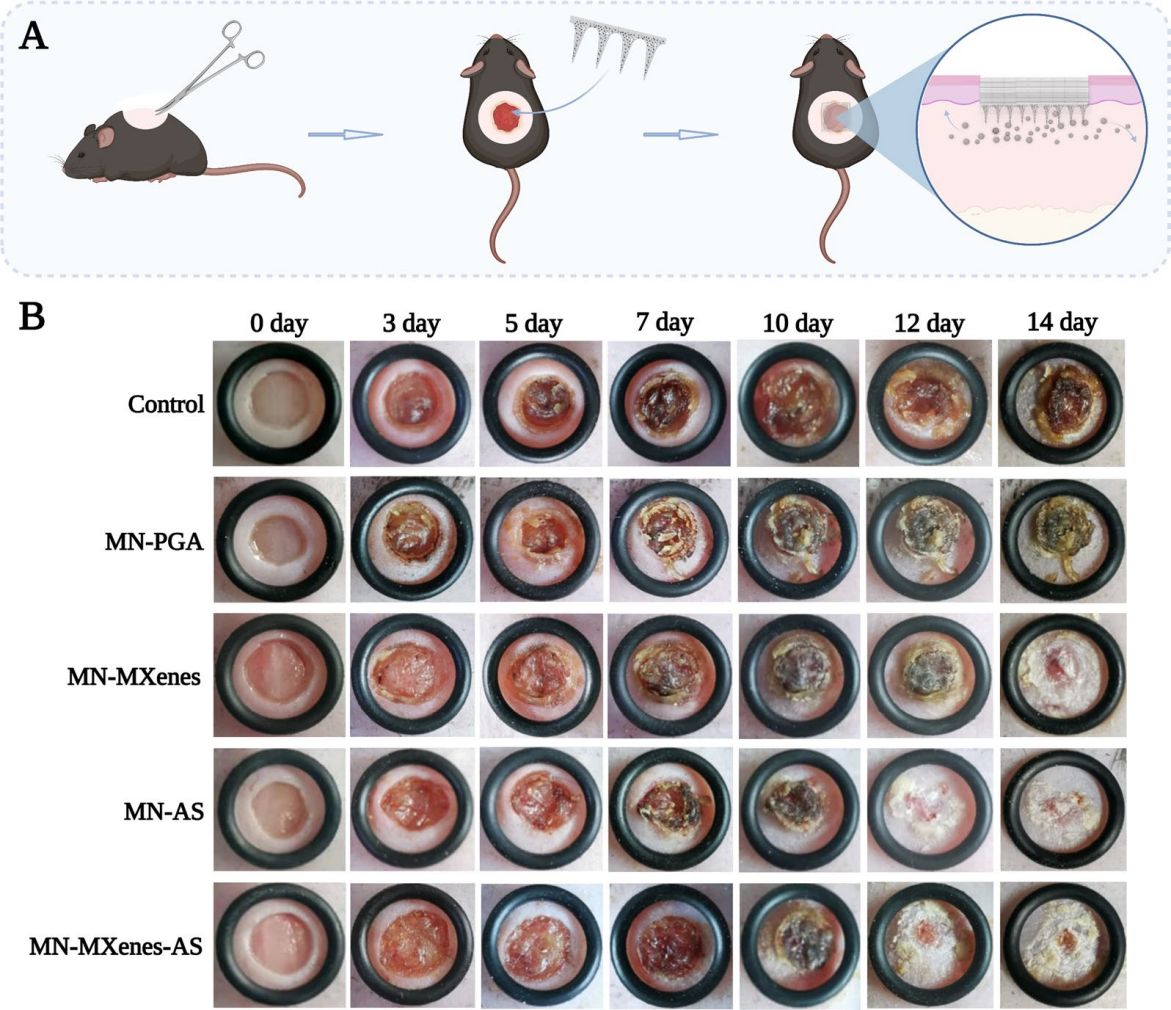
In vivo wound healing evaluation with diabetic mice. **A** Schematic illustration of MN treatment in vivo. **B** Representative photographic images of the diabetic wounds with different treatments or no treatments on 0, 3, 5, 7, 10, 12, 14 days

The in vivo diabetic wound healing efficacy of MN-MXenes-AS was tested on living diabetic mice (10-day-old). A circle wound with a 6 mm diameter was created on the back of every mouse. Wounded diabetic mice were randomly divided into five groups and treated with different microneedles (MN-PGA, MN-AS, MN-MXenes, MN-MXenes-AS or no treatment). After tips of microneedles fully were dissolved (about 20 min), the back layer was removed (Fig. 4A). To keep changes in wound areas under observation, images were taken at 0, 3, 5, 7, 10, 12, 14 days post-surgery and treatment. Fig. 4B shows that diabetic mice treated with MN-AS and MN-MXenes-AS exhibited faster and more complete wound closure than mice treated with MN-PGA or untreated mice. Therefore, the MN-MXenes-AS was an effective therapeutic agent.

### Investigation on tissue regeneration and angiogenesis after treatments

**Fig. 5:**
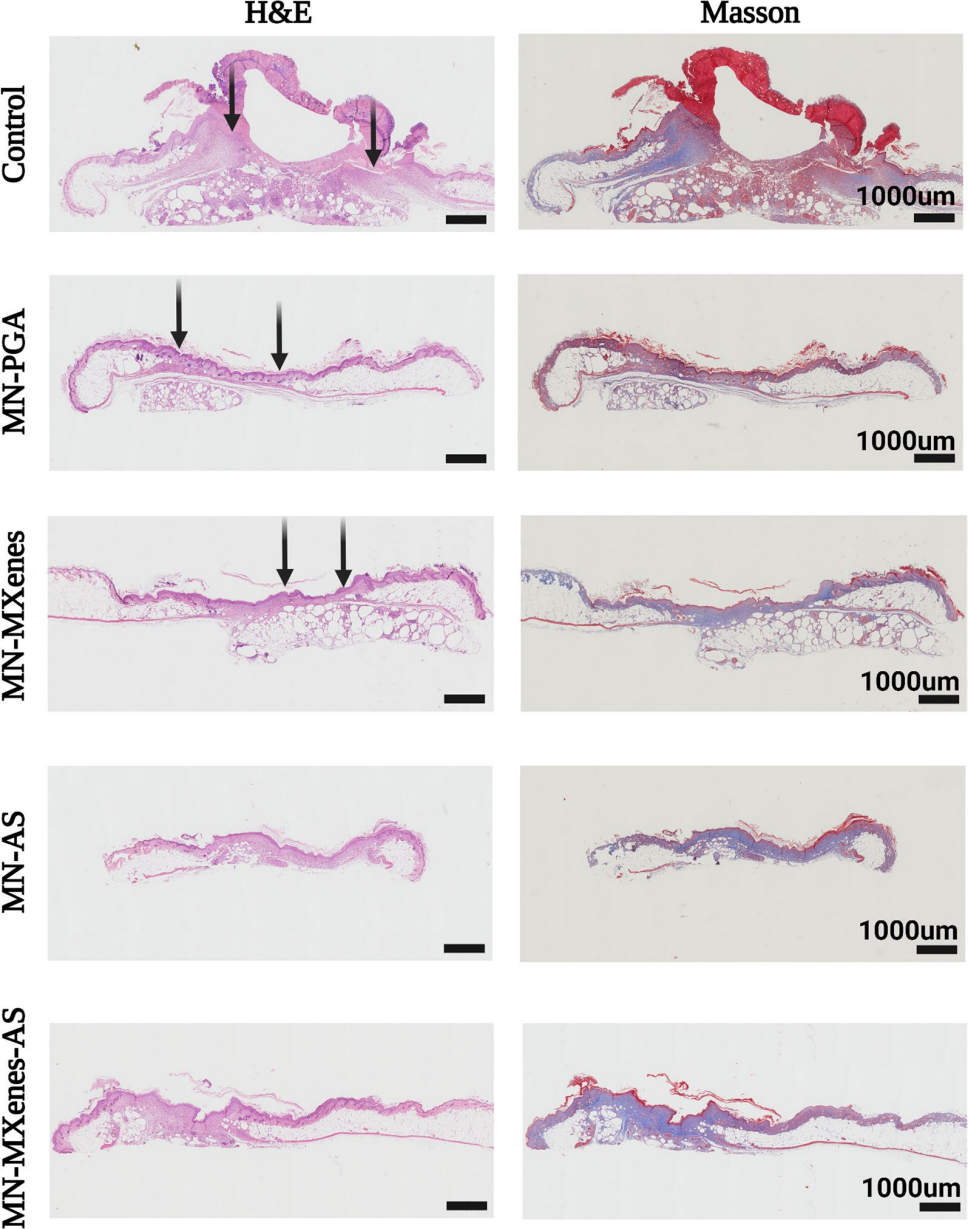
Investigation on tissue regeneration and angiogenesis after treatments. Images of H&E (first row), Masson’s trichrome (second row) staining of diabetic wounds in different groups on day 14. Black arrows indicate the edges of the wounds

**Fig. 6:**
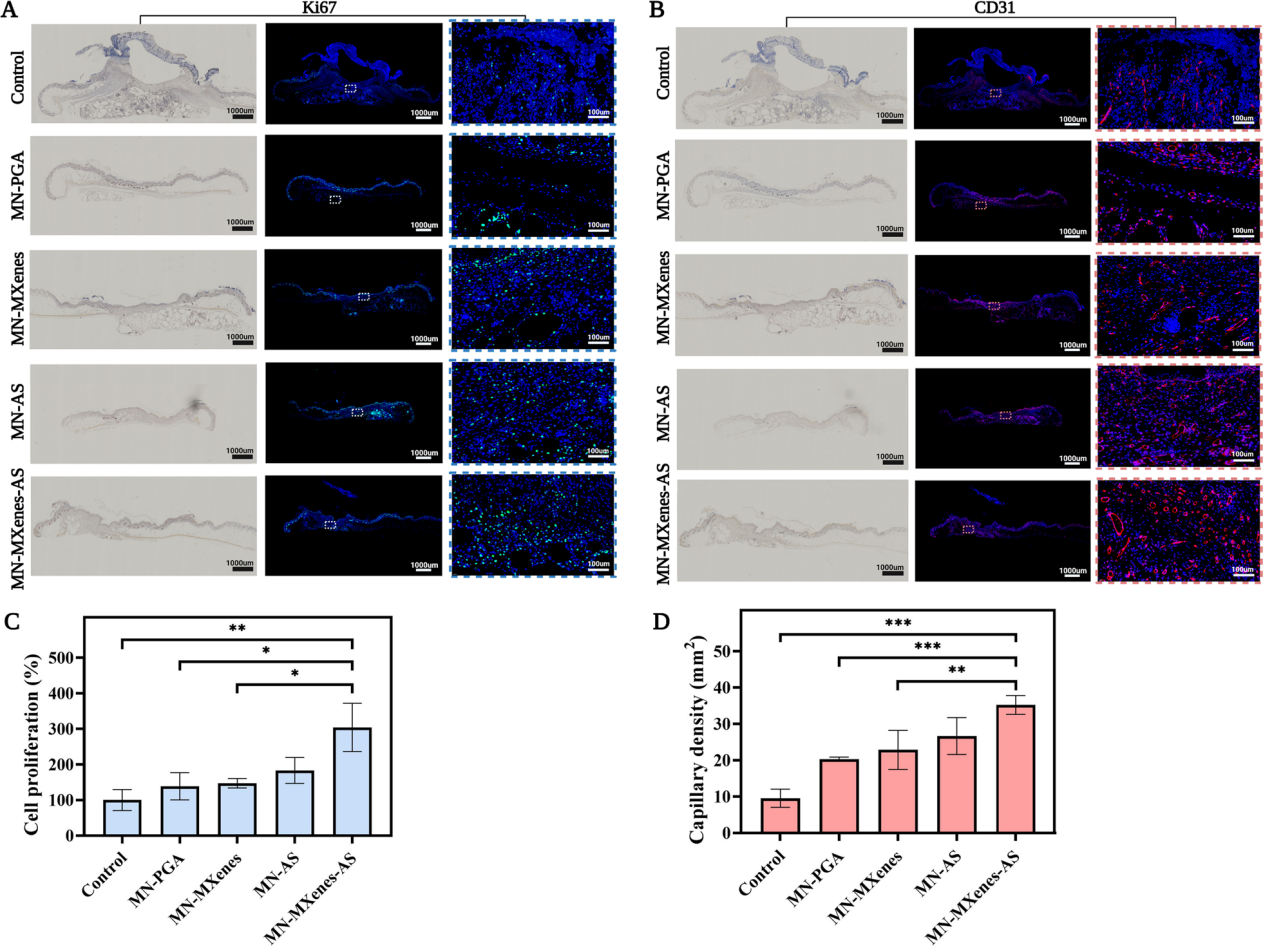
**A** Images of anti-ki67 immunofluorescence staining and anti-ki67 immunohistochemistry staining of diabetic wounds in different groups on day 14. **B** Images of anti-CD31 immunofluorescence staining and anti-CD31 immunohistochemistry staining. **C** Quantification of cell proliferation in different groups by anti-ki67 staining. **D** Quantification of capillary density by anti-CD31 staining. Rectangles indicate the magnified areas. Red arrows indicate blood vessels. n = 3, *p < 0.05, **p < 0.01, ***p < 0.001, ****p < 0.0001

Wound beds, granulation formation and epithelial formation processes were observed via haematoxylin and eosin (H&E) staining, while the collagen deposition and angiogenesis were observed using Masson’s trichrome staining (Fig. 5). The experimental groups exhibited more than twice as much angiogenesis and cell proliferation as the control group (Fig. 6C, D). Wound beds treated with MN-AS or MN-MXenes-AS exhibited more collagen formation than controls, indicating better wound closure. In addition, H&E staining and Masson’s trichrome staining tissue sections revealed an insertion depth of MN-MXenes-AS of approximately 400 μm (Additional file 1: Fig. S2).

Immunohistochemistry and immunofluorescence staining for CD31 and ki67 were used to evaluate the capillary density and cell proliferation, respectively, in different groups (Figs. 5; 6A, B). Cell proliferation rate and capillary density were quantified according to the anti-CD31 and anti-ki67 immunofluorescence staining (Fig. 6C, D). As shown in Fig. 6C, D, mice treated with MN-MXenes-AS displayed significantly more cell proliferation and higher capillary density than mice treated with MN-PGA or untreated mice.

## Discussion

DFUs have become a worldwide health problem as an increasing number of people have been diagnosed with diabetic mellitus. The most challenging problem is selecting a suitable administration method for approved therapeutics. Despite its widespread usage, smearing can be inefficient and lacks efficacy. In this study, we manufactured novel MXenes-based microneedles loaded with AS, a transdermal drug-delivering system capable of penetrating the cuticle for treating DFUs. Medical efficacy was realized through the following approaches: (1) γ-PGA, a biocompatible biopolymer, was quickly dissolved, thereby releasing the drug immersed in it; (2) enhanced mechanical strength was achieved through the incorporation of MXenes, which can function as an AS-loading system; and (3) improved epithelialisation, cell proliferation, and regulated angiogenesis were observed.

During our characterization of MN-MXenes-AS, we found that MXenes have outstanding biodegradability. In addition, compared with the fragile MN made of γ-PGA hydrogel, MN-MXenes-AS possesses better mechanical strength which makes it much easier to preserve morphological characteristics and penetrate the cuticle, thereby attaining improved insertion depth. Meanwhile, the presence of MXenes prolongs the release of AS. Importantly, the increased mechanical strength does not increase degradation time; instead, MN-MXenes-AS exhibited biodegradability, dissolving within 20 min (at 75% humidity, 25 ℃) which is quicker than organic framework-based MN we used in our previous study [25].

Several studies have used scaffolds to load different drugs for promoting epithelization and vascularization [41]. Previous studies have proved that AS has a biphasic effect [38] by differentially influencing angiogenesis at different wound-healing stages. AS inhibits the translocation of NF-κB p65 from cytoplasm to nucleus [42] and prevent increased permeability of vessel [43] by downregulating the expression of TNF-α. To promote fibroblast proliferation, several genes including LOX and LOXL3, COL1A2, COL3A1, TIMP1, CHI3L2 and ADAMTS5 would be upregulated or downregulated by AS in a dynamic way [44]. Despite the emphasis on the proliferative effect of AS in this study, its efficacy should be further investigated in a future study.

Based on the experimental outcomes presented above, groups interacting with MN-MXenes-AS possessed better angiogenesis and quicker regeneration than control groups. Collectively, due to its outstanding therapeutic efficacy, MN-MXenes-AS provides a promising subcutaneous administration mode for patients with DFUs.

## Conclusion

Herein, we manufactured a novel microneedle with sufficient rigidity to penetrate the cuticle, which promoted diabetic wound healing by increasing cell proliferation and influencing angiogenesis. Microneedles made of γ-PGA were integrated with MXenes-AS to achieve improved mechanical strength and medical value. Multifunction, biocompatibility and treatment efficacy were systematically tested both in vitro and in vivo. Overall, MN-MXenes-AS was shown to be a multifunctional subcutaneous drug-delivering system for accelerating diabetic wound healing.

## Methods

### Chemicals and materials

γ-PGA (Mw = 1000–15,000 g/mol) was purchased from Sai Taisi Biological Technology Co., Ltd. (China). Asiaticoside (AS) was bought from Aladdin (China). Indocyanine green (ICG) was purchased from AUSIA BIOTECH (Hangzhou, China). Microneedle patch moulds were made of polydimethylsiloxane (PDMS) purchased from Micropoint Technologies Pte. Ltd. (Singapore). Each microneedle had a 10 × 10 array, which was 200 × 200 × 500 μm (W × L× H). DMEM cell culture medium and foetal bovine serum (FBS) were purchased from Gibco (Carlsbad, CA, UK).

### Fabrication of MXenes-AS hydrogel

First, AS was loaded onto MXenes. 1 mL Ti_3_C_2_ MXenes dispersion (1 mg/mL) in ddH_2_O was added into 1 mL AS (2 mg/mL) solution. For better combination, the mixture was sonicated for 10 min and then stirred for 24 h [32]. Second, to produce a homodisperse MXenes-AS hydrogel, 350 mg γ-PGA was dissolved in 600 μl ddH_2_O containing 10% (60 μl) MXenes-AS. The mixed hydrogel was then ultrasonicated for 30 min at 4 °C using an ultrasonic cleaner (SK 1200BT, KUDOS, Shanghai, China).

### Fabrication and characterization of MN patch

Approximately 40 μl MXenes-AS hydrogel was added to a microneedle mold and fully filled into the tips of microneedle under vacuum for 5 min (YB-FD-1, Shanghai, China). Overflowing hydrogel resulting from vacuumizing was wiped away with spearhead followed by the addition of an appropriate amount of MXenes-AS hydrogel to the backing layer, such that the microneedle would be a suitable thickness. Subsequently, the filled microneedle mould was put into a drying baker for at least 40 min under 40 ℃ until the microneedle reached a certain degree of hardness. Then the microneedle was placed in a minipump for drying and better preservation. The fabrication of microneedle made of AS hydrogel (MN-AS), MXenes hydrogel (MN-MXenes) and only γ-PGA hydrogel (MN-PGA) follow the same technological process. Finally, microneedles were demolded and analyzed under a microscope (Nikon ECLIPSE E 100, Nikon Corporation, Japan) to observe and compare their morphology with each other.

### Hygroscopicity test of the microneedle patches

Dried microneedle patch was placed in a sealed tank at room temperature (25 ℃) with a humidity of 75%. Patches were photographed with an electron microscope (NeoScope JCM-5000) every 5 min to record the change in microneedle hygroscopicity.

### Cell isolation and culture

Fibroblasts were isolated from the back skin of a 10-day-old mouse. Mice were euthanised with an overdose of 4% chloral hydrate. For sterilization, mice were immersed in 75% ethyl alcohol for 10 s, followed by disinfection of the back skin with iodine five times. After that, the back skin was cut off, gently minced and digested with 50 mL type I collagenase (0.2% of the cell culture medium) for 2 h at 37 ℃. Finally, the mixture was centrifuged at 1500 rpm for 5 min (37 ℃) to pellet the fibroblasts. Fibroblast were then cultivated in high-glucose DMEM supplemented with 10% FBS in an incubator (37 ℃, 5%CO_2_). HUVECs were cultured under the same conditions.

### CCK-8 assay

The CCK-8 (CCK-8; Dojindo Molecular Technologies, Rockville, MD, USA) was used to examine the cytotoxicity of MXenes-AS. Fibroblast were placed in 96-well plates at 1000 cells per well and separated into five groups. Then, they were cocultured with either 100 μl cell culture medium or 90 μl cell culture medium mixed with 10 μl MN-PGA extract, MN-MXenes extract or MN-MXenes-AS extract. 10 μl cck-8 solution mixed with 90 μl cell culture medium was added to each well and cells were then placed in an incubator for 2 h (37 ℃, 5%CO_2_). Cell viability was assessed at day 3. Finally, the optical density (OD) values of all 96-well plates were measured using a microplate reader (SpectraMAX iD3, Molecular Devices. LLC., USA), and the results were analysed using GraphPad. The experiment for testing the optimal concentration of AS followed the same process, except that fibroblasts were co-cultured with different concentrations of AS solution (0, 5, 15, 30, 45, 60, 80 μg/ml).

### Living/dead cell double staining

HUVECs were seeded in confocal dishes at 40 × 10^4^ cells per dish. Cells were divided into five groups and co-cultured with either MN-PGA, MN-MXenes, MN-AS and MN-MXenes-AS, or no materials for 24 or 72 h. The Calcein-AM/PI assay (Yeasen, Shanghai) was performed according to the manufacturer’s protocol. 300 μl staining assay was added to every confocal dish and dishes were put in an incubator (37 ℃, 5% CO_2_) for 15 min after the supernatant was removed. Cells were imaged using a confocal microscope (Leica SP5, Leica Camera AG, Germany).

### The release profile of AS from the MN-MXenes-AS

Different concentration of AS (MW: 959.12 g/mol) was dissolved in PBS and the OD values were measured to obtain the standard curve. Then, MN-AS and MN-MXenes-AS were immersed in PBS placed in dialysis bags (MW: 8000–14,000) for 13 days. The dialysate was taken from the outside of the dialysis bag every day, together with dialysate of AS immersed in PBS, to determine the OD value. Subsequently, the OD value from different groups was analyzed.

### In vitro migration analysis.

Fibroblast were divided into three groups and seeded in 6-well plates. After the fibroblasts fully occupied each well, scratches were applied with spreadheads in the middle of well. Different groups of cells were cultured with AS solution (15 μg/ml), MN-MXenes-AS extract or no materials. The morphology of fibroblast was imaged with a microscope after co-culture for 0, 6, 12, and 24 h.

### Establishment of the mice model and wound efficacy testing of MN in vivo

To generate a diabetic wound model, diabetic (db/db) mice (Nanjing University-Nanjing Biomedical Institute, China) were used in this part. After the mice were anaesthetised and shaved, the back skin of each mouse was removed to create a circular wound with a diameter of 6 mm. Subsequently, 20 mice were randomly divided into 5 groups for different treatment (control group, MN-PGA group, MN-MXenes group, MN-AS and MN-MXenes-AS group). The control group received no treatment, whereas every mouse of other groups was treated with one patch of corresponding MN for 20 min after the tips of microneedle being fully dissolved under the tissue. Mouse were reared in separated cages and images were taken at 0, 3, 5, 7, 10, 12, and 14 days post-surgery.

### Histology and immunohistochemistry staining

On the 14th day after treatment, each mouse was sacrificed with overdosed 4% chloral hydrate. The wound skin was then removed, soaked in 4% paraformaldehyde for 24 h, embedded in wax blocks, and sliced into 5 μm thick sections for histological staining. H&E and Masson’s trichrome staining were performed to examine the formation of granulation tissue and collagen during wound healing. Other tissue sections were placed in rabbit monoclonal anti-CD31 and anti-ki67 antibodies (ab182981; Abcam, 1:300) and incubated overnight at 4 °C. For immunofluorescence staining, the sections were blocked with 10% normal serum blocking solution (3% bovine serum albumin and 0.1% Triton X-100 and 0.05% Tween-20) for 2 h at 25 °C. Washed sections were then incubated overnight with anti-CD31 or anti-ki67 at 4 °C.

### Statistical analysis

All quantitative data are presented as the mean ± standard deviation. Data were analyzed using GraphPad Prism 9.0, and the statistical significance was set at P < 0.05.

## Supplementary Information

**Additional file 1: Fig. S1** The Tyndall Effect of MXenes and AS in ddH_2_O and γ-PGA hydrogel. **Fig. S2** The insertion depth of MN-MXenes-ASin vivo. Fig.** S3** The morphology of MN-PGA and MN-MXenes-ASunder different humidity (25%, 50%, 75%, 90%) for 20 min. Fig.** S4** The morphology of MN-PGA under different temperature (25% humidity). **Fig. S5** The morphology of MN-MXenes-AS under different temperature (25% humidity).

## Data Availability

The authors declare that the main data supporting the findings of this study are available within the article and its Additional file Information. Extra data are available from the corresponding author upon request.

## Abbreviations

MN: Microneedle
AS: Asiaticoside
MN-PGA: Microneedle made of γ-PGA hydrogel
MN-MXenes: Microneedle made of MXenes-integrated γ-PGA hydrogel
MN-AS: Microneedle made of γ-PGA hydrogel combined with AS
MN-MXenes-AS: Microneedle made of MXenes-integrated γ-PGA hydrogel combined with AS
ICG: Indocyanine green
HUVEC: Human umbilical vein endothelial cells
