# Supplementary material for: MXenes-integrated microneedle combined with asiaticoside to penetrate the cuticle for treatment of diabetic foot ulcer
Source: J Nanobiotechnology. 2022 Jun 7;20:259. doi: 10.1186/s12951-022-01468-9 (PMC9172054; doi:10.1186/s12951-022-01468-9)
Supplement: Supplementary file 1 — Additional file 1: Fig. S1 The Tyndall Effect of MXenes and AS in ddH2O and γ-PGA hydrogel. Fig. S2 The insertion depth of MN-MXenes-ASin vivo. Fig. S3 The morphology of MN-PGA and MN-MXenes-ASunder different humidity (25%, 50%, 75%, 90%) for 20 min. Fig. S4 The morphology of MN-PGA under different temperature (25% humidity). Fig. S5 The morphology of MN-MXenes-AS under different temperature (25% humidity). [file 12951_2022_1468_MOESM1_ESM.docx]

Additional file information for

**MXenes-integrated microneedles combined with asiaticoside for accelerating diabetic wound healing**

*Pei Wang^1^, Yun Wang^1^, Yang Yi^2^, Yan Gong^1^, Haoran Ji^1^, Yuci Gan^1^, Fei Xie^2, *^, Jinchen Fan^3, *^ and Xiansong Wang^1, *^*

^1^ Department of Thoracic surgery, Shanghai Key Laboratory of Tissue Engineering, Shanghai Ninth People’s Hospital, Shanghai Jiao Tong University School of Medicine, Shanghai 200011, China.

^2^ Faculty of Environment and Life, Beijing University of Technology, Beijing 100124, R. P. China.

^3^ School of Materials and Chemistry, University of Shanghai for Science and Technology, Shanghai 200093, P. R. China.

***Corresponding authors:** [wonderluis@1](mailto:vincentuis@shsmu.edu.cn)26.com (X. Wang); [xiefei990815@bjut.edu.cn (F. Xie);](mailto:xiefei990815@bjut.edu.cn;) jcfan@usst.edu.cn (J. Fan).

**Additional file Figures**

1. The Tyndall Effect of MXenes and AS in ddH_2_O and γ-PGA hydrogel

To make the best of the therapeutic effect of MN, it is necessary for every ingredient to distribute homogeneously in the hydrogel. Hence, we performed the experiments for Tyndall Effect. MXenes and AS were first dissolve in ddH_2_O with the concentration of 0.5mg/ml and 1mg/ml respectively for test. Then, MXenes-AS hydrogel we used in our experiments was also investigated. As the images presented in Fig. S1, both MXenes and AS were perfectly distribute into γ-PGA hydrogel.


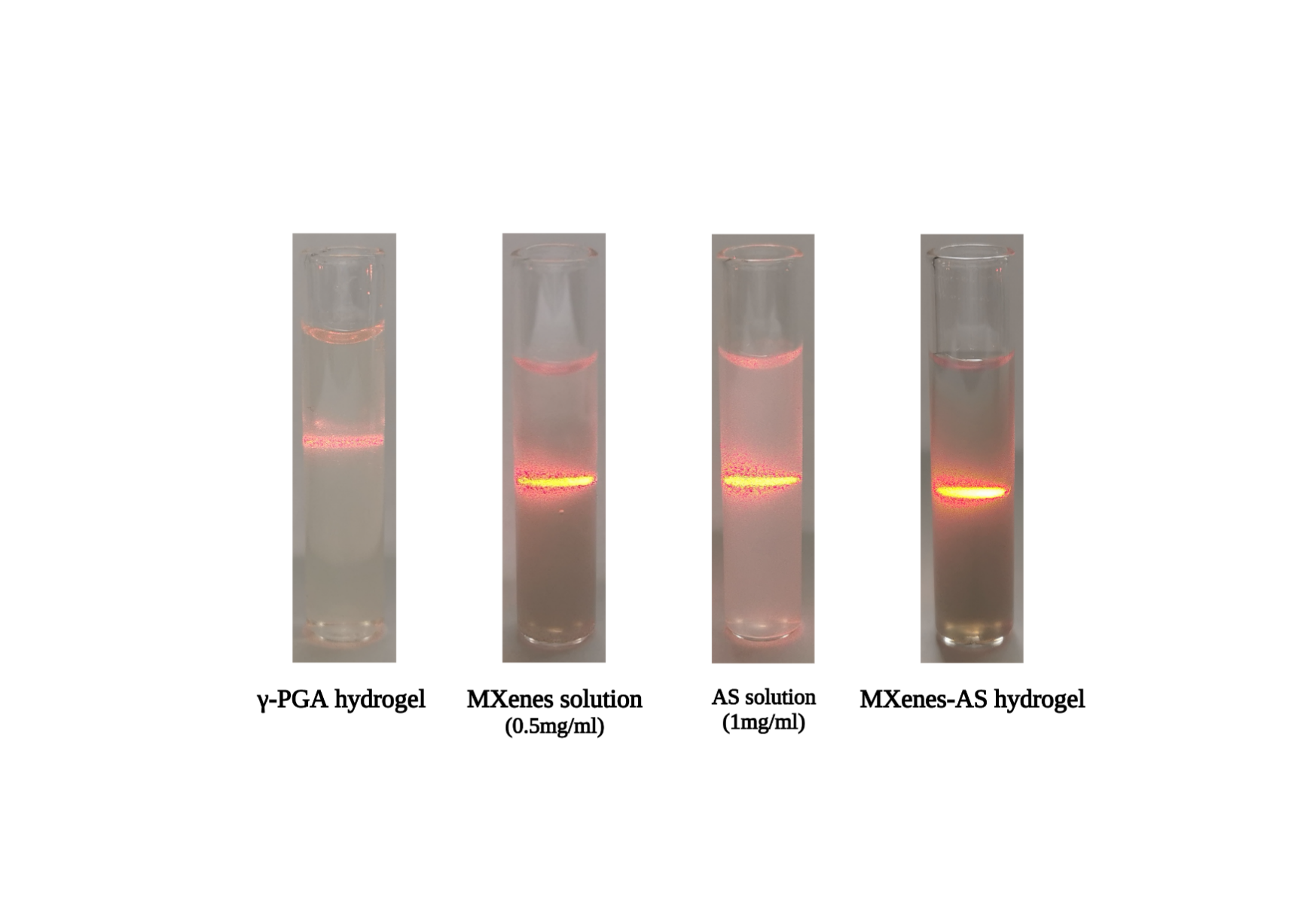


Figure S1. Tyndall Effect of γ-PGA hydrogel, MXenes solution (0.5mg/ml), AS solution (1mg/ml) and MXenes-AS hydrogel.

2. The insertion depth of MN-MXenes-AS in vivo

MN-MXenes-AS was inserted into the back of diabetic mice. According to the demonstration of H&E staining and Masson’s trichrome staining (Fig. S2). The insertion depth of MN-MXenes-AS is about 400um which means the MN has penetrated cuticle and delivered drug to the dermis.


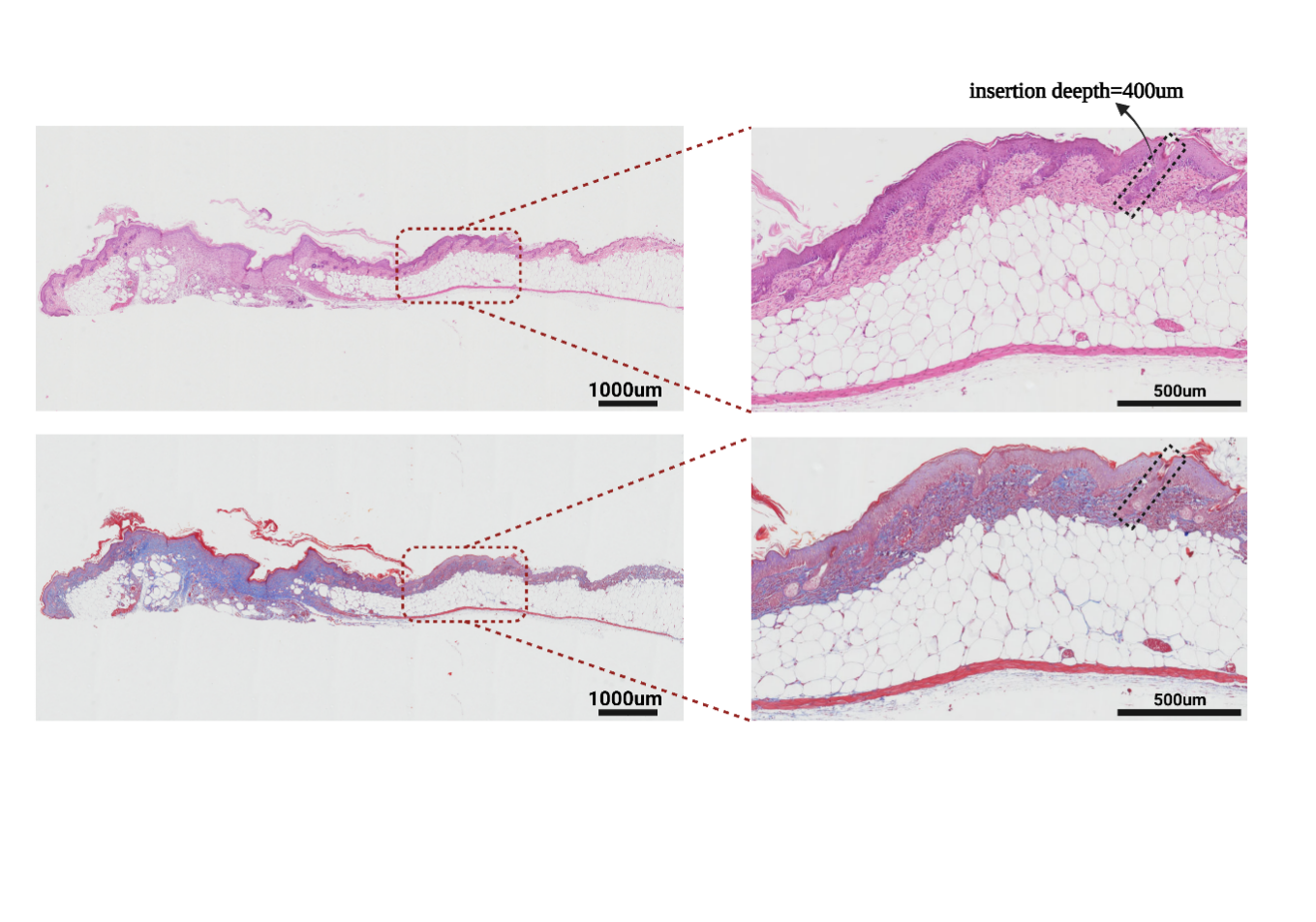


Figure S2. The insertion depth of MN-MXenes-AS in vivo

3. The morphology of MN under different humidity and temperature

To testify the morphology of MN under different humidity, MN-PGA and MN-MXenes-AS were put under the humidity of 25%, 50%, 75%, 90% for 20min when the temperature is controlled at 25℃. The SEM images were taken after that. Fig. S3 demonstrates that both MN-PGA and MN-MXenes-AS have rather good stability when the humidity is under 50%. When the humidity goes up to 75%, MN begin to dissolve. Meanwhile, despite the combination of MXenes in MN-PGA and MN-MXenes-AS, it does not affect the biodegradability of MN-PGA and MN-MXenes-AS.

To investigate the morphology of MN under different temperatures, MN-PGA and MN-MXenes-AS were placed under the temperature of -20℃, 4℃, 25℃, 50℃, 80℃ and 100℃ with controlled humidity (25%) for 1 h. Figure S4 and S5 demonstrate that the morphological characteristics of both MN-PGA and MN-MXenes-AS are rather stable under different temperatures with certain humidity, despite there are some acceptable cracks on tips of MN.

Accordingly, despite its outstanding mechanical strength, MN-MXenes-AS still possesses necessary biodegradability for in vivo application. Meanwhile, with controlled humidity, MN can be quite stable and practical for preservation.


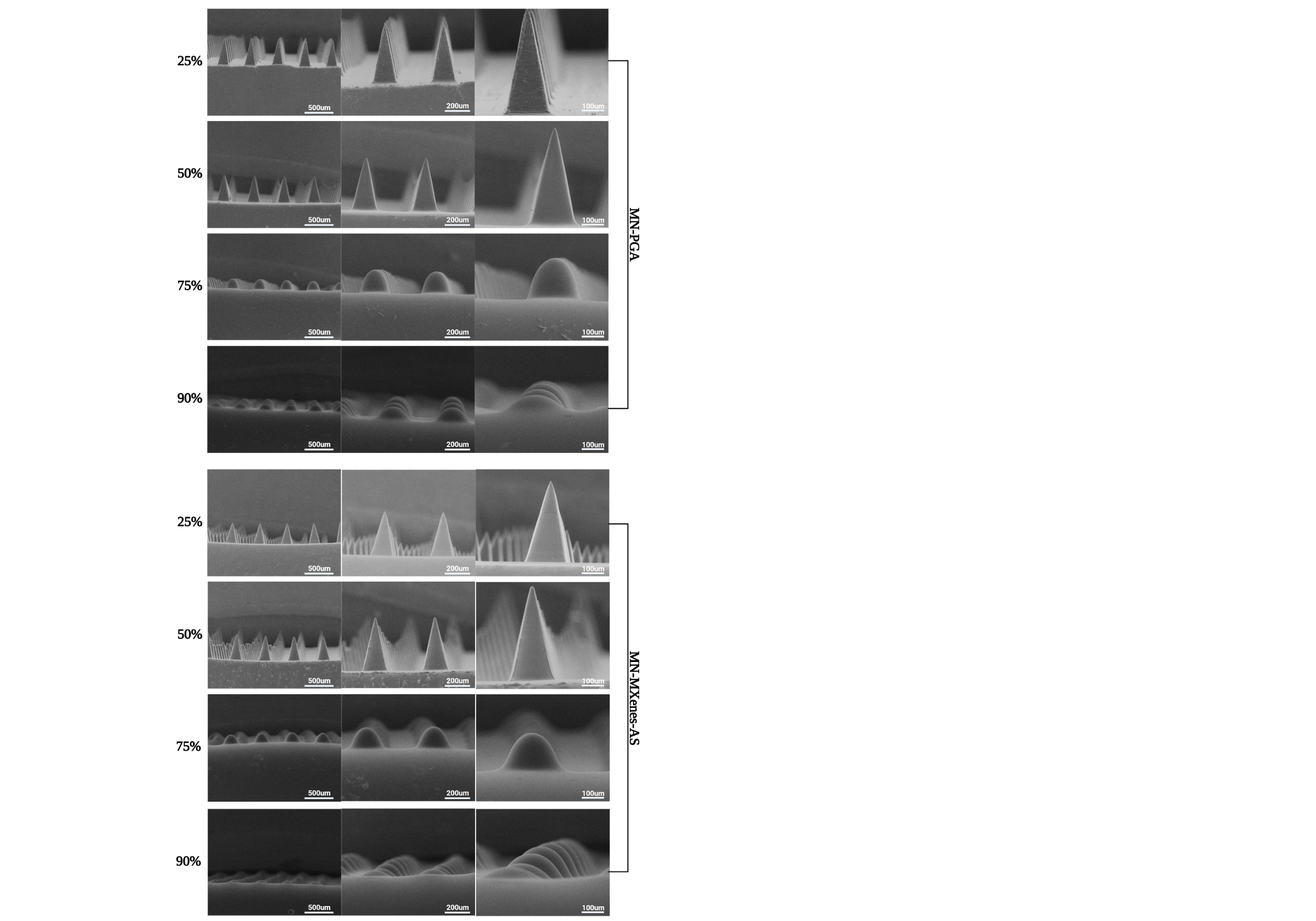


Figure S3. The morphology of MN-PGA and MN-MXenes-AS under different humidity (25%, 50%, 75%, 90%) for 20min.


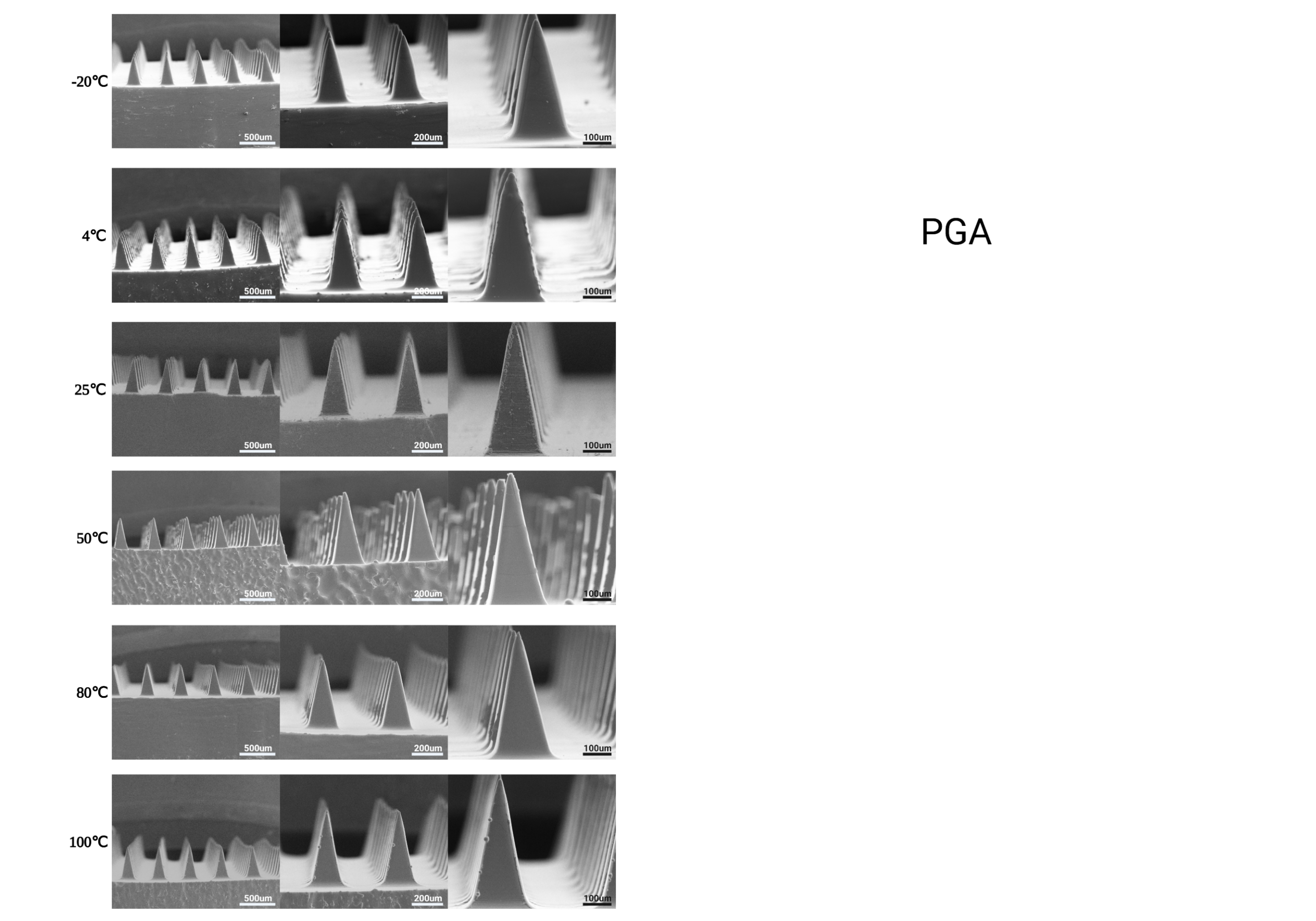


Figure 4. The morphology of MN-PGA under different temperature (25% humidity).


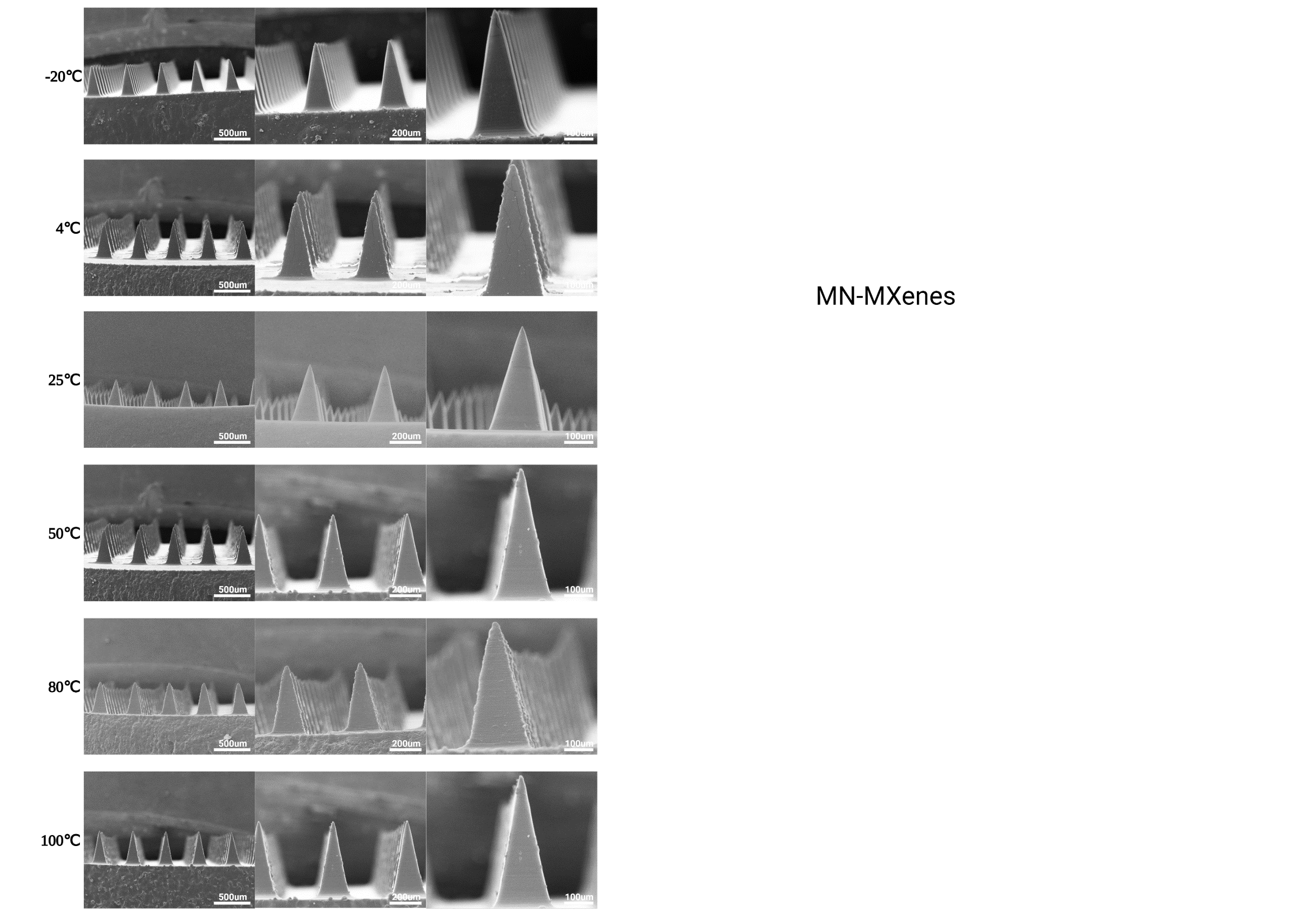


Figure 5. The morphology of MN-MXenes-AS under different temperature (25% humidity).
